# Supplementary material for: Effects of Hydrologic Regime Changes on a Taxonomic and Functional Trait Structure of Earthworm Communities in Mountain Wetlands
Source: Biology (Basel). 2023 Mar 21;12(3):482. doi: 10.3390/biology12030482 (PMC10045450; doi:10.3390/biology12030482)
Supplement: Supplementary file 1 [file biology-12-00482-s001.zip › Supplementary Table S1 Sterzynska et al.pdf]

Effects of hydrologic regime changes on taxonomic and functional trait structure of earthworm communities in mountain wetlands

Václav Pižl [1], Maria Sterzyńska [2], Karel Tajovský [1], Josef Starý [1], Paweł Nicia [3], Paweł Zadrożny [3], and Romualda Bejger[4]

Table S1. Classification of earthworm traits to the attribute classes

Species	Abb	V-D	Lmin	Lmax	Prost	Quisc	Tweek	Coc-ye	Hwidth	Disp	Higr	C/Npref	pHtol
Aporrectodea caliginosa	Acalig	3	2	3	2	3	3	3	3	1	3	1	3
Aporrectodea rosea	Arosea	3	1	3	2	3	3	3	3	3	3	1	3
Dendrobaena octaedra	Docta	1	1	2	1	1	1	1	3	1	2	3	1
Dendrodrilus rubidus	Drubid	1	1	2	1	1	1	1	3	1	2	3	1
Eisenia lucens	Eisluc	1	1	3	1	1	1	3	1	3	2	3	1
Eiseniella tetraedra	Etetra	1	1	2	1	1	1	1	1	1	1	2	3
Lumbricus rubellus	Lumrub	2	2	3	3	1	1	1	3	1	2	3	1
Octodrilus argoviensis	Octarg	2	1	2	3	1	3	3	1	2	1	3	1
Octodrilus transpadanus	Octtra	3	1	3	2	3	3	3	1	2	1	3	3
Octolasion tyrtaeum	Otyrt	3	1	3	1	3	3	3	3	3	2	2	3

Earthworm traits abbreviation

Trait	Abbreviation
vertical distribution	V-D
min. length at maturity (mm)	Lmin
max. length at maturity (mm)	Lmax
prostomium	Prost
quiescence	Quisc
time to maturity (weeks)	Tweeks
cocoons (n/year)	Coc-ye
habitat width	Hwidth
dispersal potential	Disp
hydrophily	Higr
C/N soil preference	C/Npref
low pH tolerance	pHtol
