# Supplementary material for: Effects of Hydrologic Regime Changes on a Taxonomic and Functional Trait Structure of Earthworm Communities in Mountain Wetlands
Source: Biology (Basel). 2023 Mar 21;12(3):482. doi: 10.3390/biology12030482 (PMC10045450; doi:10.3390/biology12030482)
Supplement: Supplementary file 1 [file biology-12-00482-s001.zip › Supplementary Table S2 Sterzynska et al.pdf]

Effects of hydrologic regime changes on taxonomic and functional trait structure of earthworm communities in mountain wetlands

Václav Pižl [1], Maria Sterzyńska [2], Karel Tajovský [1], Josef Starý [1], Paweł Nicia [3], Paweł Zadrożny [3], and Romualda Bejger[4]

Table S2. Earthworm density (ind. m^{-2}) in various hydrologic conditions (HC) of mountain fens

	HC	Acalig	Arosea	Docta	Drubid	Eisluc	Etetra	Lumrub	Octarg	Octtra	Otyrt
1	Natural	0.89	5.33	16.89	0.89	0.00	0.00	3.56	0.00	0.00	14.22
2	Natural	14.22	3.56	17.78	3.56	0.00	0.00	3.56	0.00	0.00	26.67
3	Natural	6.22	8.89	44.44	8.89	3.56	18.96	8.89	0.89	2.67	10.67
4	Semi-natural	0.00	0.89	38.22	4.44	1.78	9.48	0.00	16.89	0.89	8.00
5	Semi-natural	19.56	0.89	78.22	5.33	0.00	0.00	0.89	0.00	0.00	2.67
6	Semi-natural	7.11	3.56	66.67	2.67	8.89	47.41	0.89	0.00	0.89	3.56
7	Degraded	10.67	4.44	64.89	0.00	0.00	0.00	0.89	0.00	0.00	107.56
8	Degraded	11.56	1.78	50.67	4.44	0.00	0.00	0.00	26.67	0.00	5.33
9	Degraded	4.44	9.78	64.89	2.67	8.00	42.67	10.67	0.00	0.00	4.44

Earthworm species abbrevtaion

Species	Abbreviation
Aporrectodea caliginosa	Acalig
Aporrectodea rosea	Arosea

Dendrobaena octaedra	Docta
Dendrodrilus rubidus	Drubid
Eisenia lucens	Eisluc
Eiseniella tetraedra	Etetra
Lumbricus rubellus	Lumrub
Octodrilus argoviensis	Octarg
Octodrilus transpadanus	Octtra
Octolasion tyrtaeum	Olact
